# Environmental enrichment improves declined cognition induced by prenatal inflammatory exposure in aged CD-1 mice: Role of NGPF2 and PSD-95

**DOI:** 10.3389/fnagi.2022.1021237

**Published:** 2022-11-21

**Authors:** Ming-Zhu Ni, Yue-Ming Zhang, Yun Li, Qi-Tao Wu, Zhe-Zhe Zhang, Jing Chen, Bao-Ling Luo, Xue-Wei Li, Gui-Hai Chen

**Affiliations:** ^1^Department of Neurology (Sleep Disorders), The Affiliated Chaohu Hospital of Anhui Medical University, Hefei, China; ^2^Department of Neurology, The First Affiliated Hospital, Hengyang Medical School, University of South China, Hengyang, China

**Keywords:** aging, inflammation, learning and memory, environmental enrichment, NGPF2, PSD-95

## Abstract

**Introduction:**

Research suggests that prenatal inflammatory exposure could accelerate age-related cognitive decline that may be resulted from neuroinflammation and synaptic dysfunction during aging. Environmental enrichment (EE) may mitigate the cognitive and synaptic deficits. Neurite growth-promoting factor 2 (NGPF2) and postsynaptic density protein 95 (PSD-95) play critical roles in neuroinflammation and synaptic function, respectively.

**Methods:**

We examined whether this adversity and EE exposure can cause alterations in *Ngpf2* and *Psd-95* expression. In this study, CD-1 mice received intraperitoneal injection of lipopolysaccharide (50 μg/kg) or normal saline from gestational days 15–17. After weaning, half of the male offspring under each treatment were exposed to EE. The Morris water maze was used to assess spatial learning and memory at 3 and 15 months of age, whereas quantitative real-time polymerase chain reaction and Western blotting were used to measure hippocampal mRNA and protein levels of NGPF2 and PSD-95, respectively. Meanwhile, serum levels of IL-6, IL-1β, and TNF-α were determined by enzyme-linked immunosorbent assay.

**Results:**

The results showed that aged mice exhibited poor spatial learning and memory ability, elevated NGPF2 mRNA and protein levels, and decreased PSD-95 mRNA and protein levels relative to their young counterparts during natural aging. Embryonic inflammatory exposure accelerated age-related changes in spatial cognition, and in *Ngpf2* and *Psd-95* expression. Additionally, the levels of *Ngpf2* and *Psd-95* products were significantly positively and negatively correlated with cognitive dysfunction, respectively, particularly in prenatal inflammation-exposed aged mice. Changes in serum levels of IL-6, IL-1β, and TNF-α reflective of systemic inflammation and their correlation with cognitive decline during accelerated aging were similar to those of hippocampal NGPF2. EE exposure could partially restore the accelerated decline in age-related cognitive function and in *Psd-95* expression, especially in aged mice.

**Discussion:**

Overall, the aggravated cognitive disabilities in aged mice may be related to the alterations in *Ngpf2* and *Psd-95* expression and in systemic state of inflammation due to prenatal inflammatory exposure, and long-term EE exposure may ameliorate this cognitive impairment by upregulating *Psd*-*95* expression.

## Introduction

The risk of developing many pathological conditions, especially cognitive decline, increases rapidly with age. It is estimated that 40% of the population, aged 60 years or more, is affected by age-related cognitive decline (Vanguilder and Freeman, 2011). Age-related cognitive decline hampers quality of life, and, consequently, the financial cost of providing long-term care for current and future sufferers of this condition is overwhelming. Thus, there is a need to understand the mechanisms that contribute to this loss of cognitive function with normal aging. However, the aforementioned mechanisms remain largely unclear, and effective approaches to alleviate brain aging are still lacking.

Current evidence demonstrates that exposure to maternal immune activation (MIA) *in utero* in rodents causes cognitive abnormalities, such as spatial learning and memory impairments, in adult offspring (Batinić et al., 2016; Simões et al., 2018). These abnormalities are similar to those reported in human studies (Ross-Munro et al., 2020; Veerasammy et al., 2020). Lipopolysaccharide (LPS), a component of the Gram-negative bacterial cell wall, activates the immune system (Domínguez-Rivas et al., 2021). Administration of LPS in rodents leads to inflammatory responses in astrocytes and microglial cells, which subsequently produce multiple pro-inflammatory mediators, such as interleukin (IL)-6, IL-1β, and tumor necrosis factor-alpha (TNF-α; Colonna and Butovsky, 2017; Liang et al., 2019; Domínguez-Rivas et al., 2021). Pro-inflammatory mediators cross the dysfunctional placental and blood–brain barriers to enter the fetal blood circulation and brain, thereby elevating concentrations of cytokines in fetuses during and after LPS-induced MIA (Simões et al., 2018; Cadaret et al., 2019). In addition, fetuses *in utero* trigger their own inflammatory response and promote the production of inflammatory cytokines after MIA (Elovitz et al., 2011; Henrique et al., 2021). These changes have the capacity to modulate fetal neurodevelopmental trajectories, thereby impairing cognitive performance in adult offspring (Henrique et al., 2021; Guma et al., 2022). However, only a limited number of studies have investigated the age-associated cognitive consequences of prenatal MIA exposure in these offspring, especially after midlife. Our previous studies indicated that exposure to LPS-induced MIA during late embryogenesis accelerated age-related learning and memory impairment in mouse offspring, particularly at midlife to senectitude (Li et al., 2016; Wang et al., 2020; Zhang et al., 2020).

The hippocampus, a brain region critical for learning and memory, is vulnerable to the aging process (Geinisman et al., 1995). Neuroinflammation is thought to contribute to aging and age-related cognitive impairment by damaging hippocampal synaptic function (Vanguilder and Freeman, 2011; Bettio et al., 2017; Juan and Adlard, 2019). The neurite growth-promoting factor 2 (NGPF2; also referred to as midkine) is not only a cytokine but also a neurotrophic factor (Ross-Munro et al., 2020; Zhou et al., 2021). It is principally expressed in the developing central nervous system (CNS; Zhou et al., 2021) and is usually reduced to negligible level in adulthood (Yoshida et al., 2014). However, mounting evidence indicates that NGPF2 is markedly upregulated in many pathological conditions that are characterized by inflammatory injury. For example, NGPF2 was highly expressed in the cerebral peri-infarct area (Yoshida et al., 1995) and in senile plaques in the brain of Alzheimer’s disease (AD) patients (Yasuhara et al., 1993). Further investigations indicate that NGPF2 exhibits neuroprotective properties in the aforementioned diseases (Ishikawa et al., 2009; Herradón and Pérez-García, 2014), which may be beneficial for cognitive function. NGPF2 also exacerbates various pathological processes (Cai et al., 2020; Filippou et al., 2020; Ross-Munro et al., 2020). Interestingly, NGPF2 was found to specifically modulate neuroinflammation resulting from amphetamine injection and traumatic brain injury, thereby impairing cognitive and neurological consequences (Vicente-Rodríguez et al., 2016; Takada et al., 2020). Briefly, NGPF2 is also a regulator of cognitive impairment. Various research has revealed the “double-edged sword” effect of NGPF2, due to its ability to repair nerve damage or aggravate injury outcomes depending on different pathological factors, including age, brain region, disease state, and stimuli (such as amphetamine and LPS; Yoshida et al., 2014; Vicente-Rodríguez et al., 2016; Fernández-Calle et al., 2018). We hypothesized that the “double-edged sword” effect of NGPF2 may impair or protect cognition in specific conditions. Therefore, we aimed to investigate how inflammatory exposure during late embryogenesis changes *Ngpf2* gene expression in the brain at different ages, and whether NGPF2 is associated with age-related impairment in spatial learning and memory that is caused by embryonic MIA exposure.

Additionally, studies have demonstrated chronic and systemic immune activation in offspring following prenatal exposure to MIA (Hsiao et al., 2012; Rose et al., 2017). Human and animal studies suggest that systemic inflammation reflected by peripheral inflammatory cytokines may be linked to the exacerbation of neurodegenerative diseases such as AD and cognitive decline in older adults (Walker et al., 2019; Ashraf-Ganjouei et al., 2020). However, it is unknown whether MIA exposure *in utero* increases systemic inflammation in aged offspring.

The disruption of hippocampal synaptic function contributes to age-related cognitive decline (Petralia et al., 2014; Juan and Adlard, 2019). Indeed, synaptic deficits observed in the hippocampus may be partially explained by alterations in the level of postsynaptic density protein 95 (PSD-95; Bettio et al., 2017; Juan and Adlard, 2019). PSD-95 is a critical synaptic protein that regulates synaptic plasticity (Dore et al., 2021) and supports the stability of hippocampus-dependent memory (Fitzgerald et al., 2015). PSD-95 is reduced in the hippocampus of an AD mouse model (Shao et al., 2011; Xiao et al., 2021), as well as in aged rats with cognitive impairment when compared to aged cognitively unimpaired or young rats (Savioz et al., 2014). Neuroinflammation and other pathologies could reduce hippocampal *Psd-9* expression (Savioz et al., 2014). As mentioned, NGPF2 is induced after inflammatory injury and may also aggravate various pathologies. Although many studies have involved PSD-95, there are only limited reports on whether embryonic inflammatory exposure alters hippocampal *Psd-95* expression in aged mice.

In contrast, studies in rodents suggest that environmental enrichment (EE) may increase PSD-95 level in the brain (Savioz et al., 2014; Griñán-Ferré et al., 2018). It is well-known that rodents exposed to EE, either sensory or social, and physical exercises (i.e., larger group and larger cage equipped with additional running wheels, tunnels, ladders, and toys) have higher levels of brain-derived neurotrophic factor and improved inflammation and synaptic plasticity in the hippocampus (Griñan-Ferré et al., 2016a,b; Bettio et al., 2017). Thus, EE has been proposed as a non-invasive mean to ameliorate spatial learning and memory deficits (Wang et al., 2018; Kubo et al., 2021). However, it remains elusive whether EE is sufficient to mitigate the detrimental effects of prenatal inflammatory exposure and normal aging on age-related learning and memory decline, as well as the possible concomitant changes in *Psd*-95 and N*gpf2* expression, or systematic inflammation.

In this study, the effects of prenatal inflammatory exposure and EE treatment after weaning on spatial learning and memory were investigated in young and aged CD-1 mice. Additionally, whether mRNA and protein levels of NGPF2 and PSD-95 in the hippocampus and the levels of serum inflammatory cytokines differed among the different treatment groups, including both young and old mice, were examined. Finally, correlations between measured neurobiological indicators and cognitive performance were determined.

## Materials and methods

### Animals and experimental protocol

**Figure 1:**
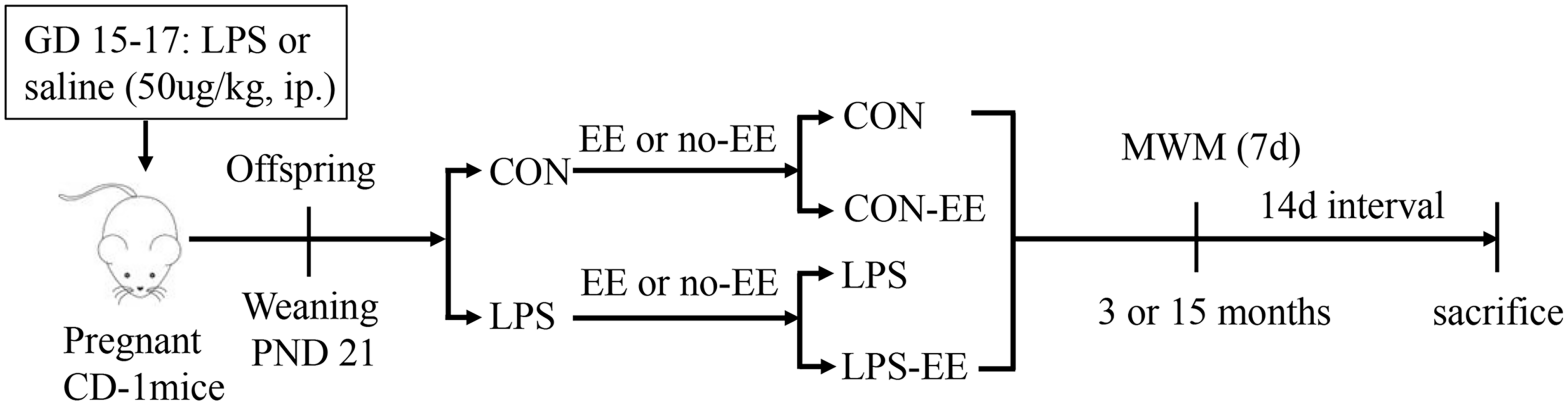
Timeline of experimental events. Pregnant mice were intraperitoneally injected with LPS or normal saline daily on days 15–17 of gestation (GD). All male offspring were weaned at postnatal day (PND) 21 and were divided into four groups (CON, CON-EE, LPS, and LPS-EE). Morris water maze tests were performed at 3 and 15 months of age. Fourteen days after testing, mice were sacrificed for subsequent biochemical experiments. CON, untreated control group; LPS, lipopolysaccharide treatment group; EE, group of mice exposed to environmental enrichment; MWM, Morris water maze.

CD-1 mice (4–6 weeks old) were purchased from Beijing Vital River Laboratory Animal Technology Co. Ltd. The animals were housed in standard plastic mouse cages (31.8 × 20.2 × 13.5 cm^3^) and maintained under standard laboratory conditions, with a constant temperature of 22°C–25°C and 55 ± 5% humidity on a 12-h light–dark cycle (lights on at 07:00). Food and water were available *ad libitum*. After 2 weeks of adaptive feeding, female mice were paired with males at a 2:1 ratio. On the next morning, the emergence of a vaginal plug was considered as gestational day (GD) 0. From GDs 15–17, the pregnant mice received a daily intraperitoneal injection (i.p.) of LPS (50 μg/kg) or the same volume of normal saline, as previously described (Wang et al., 2020; Zhang et al., 2020). The offspring were raised by their mother until weaning at postnatal day 21. The male pups were assigned to one of four groups: (1) the control (CON) group—whose mother received i.p. saline; (2) the LPS group—whose mother received i.p. LPS; (3) control exposed to EE treatment group (CON-EE); and (4) LPS exposed to EE treatment group (LPS-EE). Specifically, half of the male pups in each litter whose mothers received LPS ip were randomly assigned to the LPS group and the other half were assigned to the LPS-EE group, and this assignment rule was also followed for male pups whose mothers received saline ip. CON and LPS groups were assigned to the same mouse cages (4–5 mice per cage) as their mothers. The other two groups exposed to the EE were housed in larger cages (54.5 × 39.5 × 20 cm^3^, 8–10 mice per cage) equipped with additional toys, including running wheels, tunnels, ladders, bridges, and log cabins, which were used to trigger cognitive and physical activities and social interactions (Ismail et al., 2021). Mice were exposed to novelty stimulation by renewing toys daily, and the program cycled weekly. EE exposure (short-term EE: from weaning to 3 months of age; long-term EE: from weaning to 15 months of age) continued until the end of the behavioral experiment. When the mice reached 3 and 15 months of age, 6 mice from each group were randomly selected for the experiment. The experimental schedule is shown in Figure 1. All experimental procedures complied with the guidelines established by the National Institutes of Health Guide for the Care and Use of Laboratory Animals, and were approved by the Experimental Animal Ethics Committee of Anhui Medical University (No. LLSC20160165).

### Morris water maze

The Morris water maze (MWM) is a widely used task for evaluating hippocampus-dependent spatial learning and memory (Domínguez-Rivas et al., 2021). Our apparatus consisted of a circular tank (150 cm diameter, 30 cm height) with a black inside wall and an escape platform (10 cm diameter, 24 cm height). The tank was placed on a secure steel frame with a height of 30 cm and was filled with water (20°C–22°C, depth of 25 cm). The pool was divided into four quadrants, and the escape platform was placed 1 cm below the water’s surface in the center of one of the quadrants. The periphery of the pool was surrounded by a white curtain, forming a cylindrical shape from the ceiling to the ground. Three clearly seen black geometric figures (circles, squares, and triangles) were equidistantly suspended inside the drapery, 150 cm above the ground, to serve as spatial cues. The mice to be tested were moved to the behavioral assessment room for acclimatization 3 days prior to testing. The day before the experiment, the mice were trained to find platforms placed in different positions, to assess mice for adequate vision and swimming ability. The platform had a flag and was 1 cm above the water’s surface. During training or acquisition trials (learning phase), the mice were tested four times per day, with 15-min intervals between trials, for 7 days. On day 1, the mice were placed on the escape platform for 30 s before the first trial began. Subsequently, mice were randomly placed into the water from different quadrants (except the platform quadrant) facing the pool wall. Mice were allowed to spend 60 s to reach a hidden escape platform and rest on the platform for 30 s in each trial. If they failed to find the platform within 60 s, they were gently guided to the platform for 30 s. On the last day, the mice underwent probe trials (memory phase) with the platform removed. Two hours after the last learning trial, the mice were placed in the quadrant opposite the platform position and allowed to swim for 60 s. All the parameters of the swimming velocity, distance, and time were recorded using Any-maze software (Stoelting, United States). Experiments were conducted by fixed experimenters at the same time of day.

The time that mice take to reach an escape platform (escape latency) is the most widely used measure of learning ability. However, escape latency can be affected by the swimming speed of mice. Because the swimming distance can better reflect the learning ability of aged mice in the learning phase (Zhang et al., 2020), the swimming distance was preferentially used as an indicator of learning performance of mice in the present study, and the percentage of swimming distance and time in the target quadrant was recorded to evaluate the level of memory consolidation, as per previous studies (Wu et al., 2020; Zhuang et al., 2021).

### Tissue and serum preparation

To avoid the possible effects of previous experimental manipulations on mRNA or protein level, the mice were sacrificed by cervical dislocation and decapitated 2 weeks after the behavioral tests. Retro-orbital blood samples were collected, and serum was subsequently separated by centrifuging samples at 4,000 rpm for 6 min. Serum was stored at −80°C. The hippocampus was isolated and immediately stored at −80°C for quantitative real-time polymerase chain reaction (qRT-PCR) and Western blotting.

### Serum inflammatory cytokine measurement

Serum concentrations of IL-6, IL-1β, and TNF-α were determined by enzyme-linked immunosorbent assay using IL-6, IL-1β, and TNF-α kits, respectively (Wuhan ColorfulGene Biological Technology Co., Ltd., China).

### qRT-PCR

qRT-PCR was conducted as previously reported (Zhuang et al., 2021). Total RNA was extracted from the hippocampus using Trizol reagent according to the manufacturer’s instructions. The purity and content of total RNA were determined using a spectrophotometer. The PrimeScript™ RT reagent Kit (Takara, RR047A) was used for reverse transcription of total RNA to complementary DNA (cDNA). Transcripts were amplified by qRT-PCR using Novostart SYBR qPCR SuperMix Plus in a 10 μl total reaction mixture (5 μl of 2× SYBR Green mixture, 1 μl of each primer (10 μM), 1 μl of cDNA template, and 2 μl of RNase-free water). qRT-PCR was performed under the following conditions: one cycle of 95°C for 1 min, 40 cycles of 95°C for 20 s, and 60°C for 1 min. mRNA levels were quantified using the 2^△△Ct^ method. Beta-actin was used as the endogenous control, and the primer sequences are listed in Table 1.

**Table 1:** Primer sequences used for qRT-PCR.

**Gene**	**Forward primer (5′ → 3′)**	**Reverse primer (5′ → 3′)**
β-actin	AGTGTGACGTTGACATCCGT	TGCTAGGAGCCAGAGCAGTA
NGPF2	CTGAGACATCGGTTCCAAGT	ATCTTGTCCCACTTTCCAGG
PSD-95	CCCAGGATATGTGAACGGAA	CCTGAGTTACCCCTTTCCAA

### Western blotting

Hippocampal tissue was lysed in RIPA lysis buffer (Beyotime, China) and centrifuged at 12,000 rpm for 15 min at 4°C. The supernatant was extracted and mixed with 5× sodium dodecyl sulfate polyacrylamide gel electrophoresis (SDS-PAGE) protein loading buffer and boiled for 15 min. An equal amount of total protein from each sample was separated by 10% SDS-PAGE and transferred onto polyvinylidene fluoride immunoblotting membranes (Millipore, United States). Membranes were blocked with nonfat powdered milk in Tris-buffered saline with Tween-20 (TBST) for 2 h at 25°C, and then incubated overnight with primary antibodies to rabbit NGPF2 (1:1,000; ab52637, Abcam), rabbit PSD-95 (1:2,000; ab238135, Abcam), and mouse GAPDH (1:1,000; TA-08; Zsbio, China) at 4°C. After washing three times with TBST, the membranes were incubated with horseradish peroxidase-conjugated secondary antibodies (1:12,000; ZB2301, ZB-2305; Zsbio, China) for 1.2 h at room temperature. After rinsing again, the immunoreactive bands for the protein of interest were visualized using an enhanced chemiluminescence reagent (Thermo, United States). Immunoreactive bands revealed positive expression at 16 kDa (NGPF2), 80 kDa (PSD-95), or 36 kDa (GAPDH, internal standard). The quantification of band density was analyzed using ImageJ. Duplicate samples were averaged for each subject. All quantitative analyses were normalized to the corresponding GAPDH control.

### Statistical analysis

All data with normal distribution are presented as the mean ± standard error of the mean (SEM). For data with normal distribution and equal variance, Student’s *t*-test, one-way analysis of variance, or mixed-effects model with repeated measures and *post hoc* Tukey’s tests for multiple comparison were used as appropriate. Specifically, data reflecting performance in the MWM learning task were analyzed using mixed-effects model with repeated measures. To determine the main effects of treatment, the other data were analyzed by one-way analysis of variance. The student’s *t*-test was used to analyze the effect of age. Correlations between MWM performance and target mRNA or protein level in the hippocampus were analyzed using the Pearson’s correlation coefficient test. All analyses were performed using GraphPad Prism 8 Software, and *p* < 0.05 was considered to be statistically significant.

## Results

### Performances in the Morris water maze

#### Age effects

**Figure 2:**
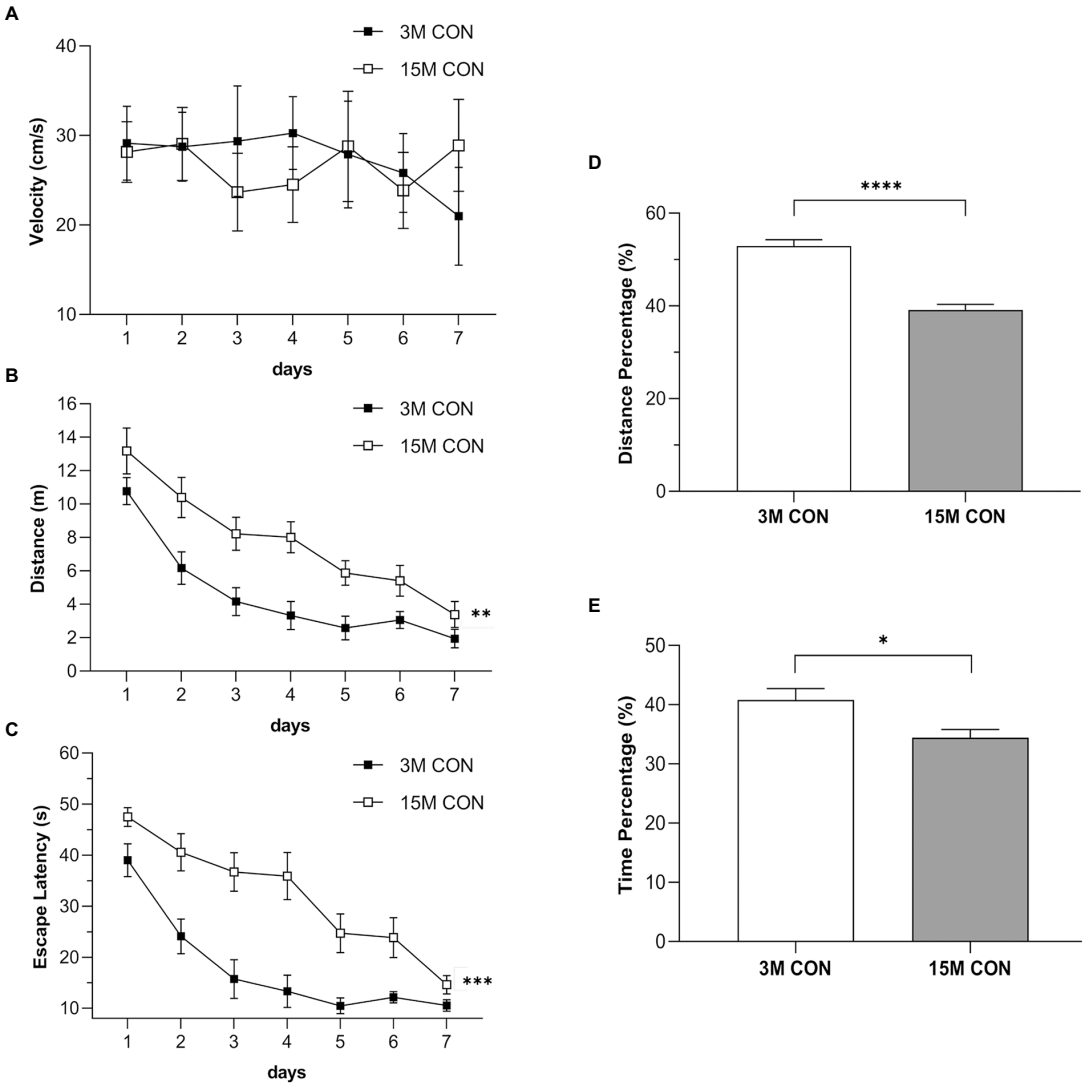
Performances illustrating the age effects in the Morris water maze test. Swimming velocity **(A)**, distance swam **(B)** and escape latency **(C)** during the training phase, and the percentage of distance **(D)** and time **(E)** swam in the target quadrant during the memory phase in the CON group at 3 months (3 M) and 15 months (15 M) of age. Data are presented as means ± SEM (*n* = 6 male mice/group). ^**^p < 0.01, ^***^p < 0.001 compared with the CON group; CON, untreated control group.

Results of swimming velocity in the CON mice (Figure 2A) indicated that age did not significantly alter motor performance of aged mice. The distance swam (Figure 2B) and escape latency (Figure 2C) of the CON group gradually decreased with the increase in training days. Furthermore, age significantly affected learning and memory functions. Compared to the 3-month-old CON mice, the15-month-old CON mice had longer distance swam (Figure 2B) and latency (Figure 2C) during the training phase and lower percentage of distance (Figure 2D) and time (Figure 2E) swam in the target quadrant during the memory phase.

#### Treatment effects

##### During the acquisition phase

**Figure 3:**
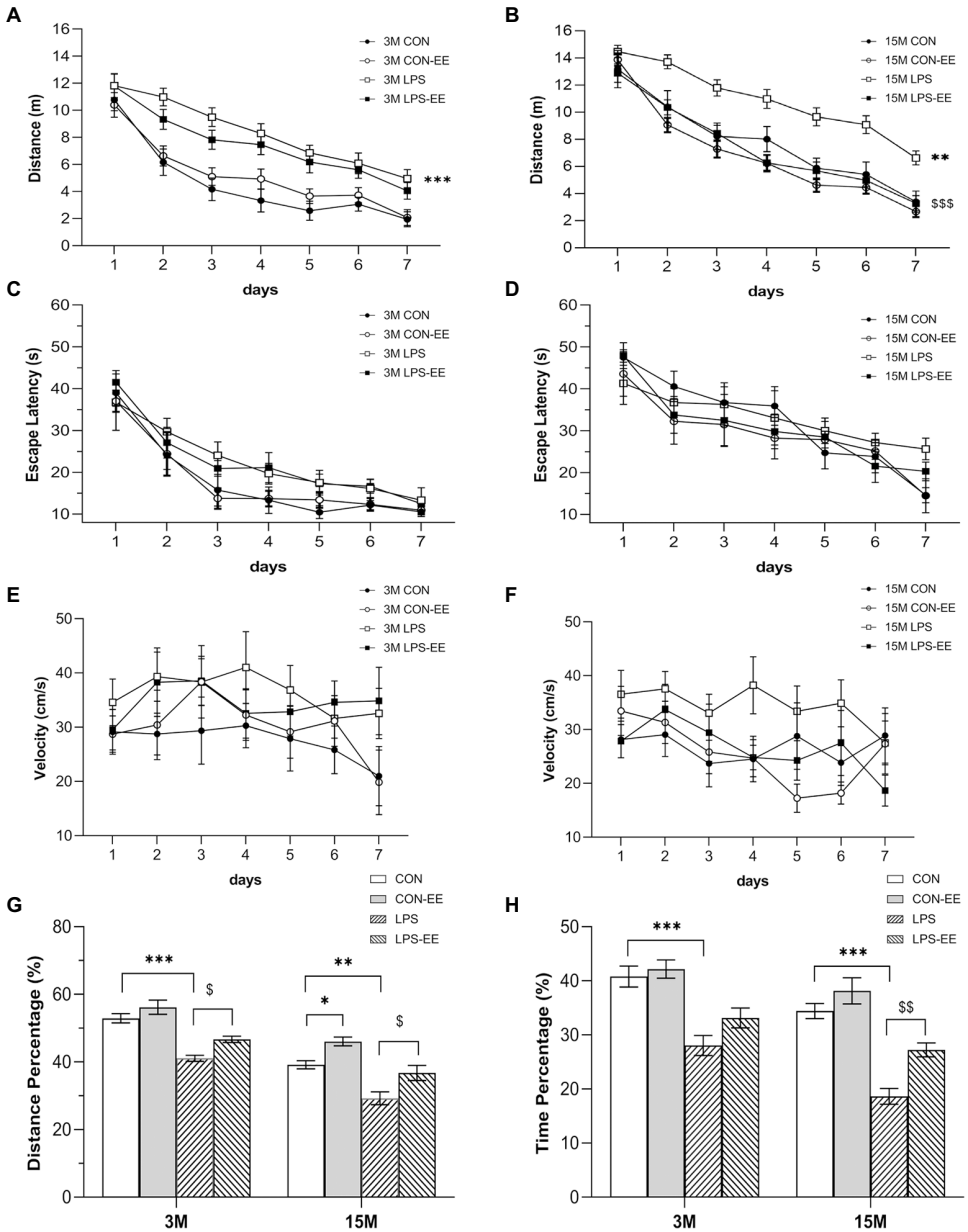
Performances in the Morris water maze test with different treatments. Distance swam **(A,B)**, escape latency **(C,D)**, and swimming velocity **(E,F)** during the learning phase, and percentage of distance **(G)** and time **(H)** swam in the target quadrant during the memory phase in the different treatment groups at 3 months (3 M) and 15 months (15 M) of age. All data are shown as means ± SEM (*n* = 6 male mice/group). ^*^p < 0.05, ^**^p < 0.01, ^***^p < 0.001 compared with the CON group; ^$^*p* < 0.05, ^$$^*p* < 0.01, ^$$$^*p* < 0.001 compared with the LPS group; CON, untreated control group; LPS, lipopolysaccharide treatment group; EE, group of mice exposed to environmental enrichment.

The effects of treatment were significant for the distance swam (Figures 3A,B) but not the latency (Figures 3C,D) or swimming velocity (Figures 3E,F) in the different treatment groups at both 3 and 15 months of age. At 3 months of age, the LPS group exhibited a significant spatial learning disability, with a greater distance swam, as compared with the CON group (Figure 3A). However, the short-term EE exposure did not significantly affect learning ability (CON vs. CON-EE; LPS vs. LPS-EE; Figure 3A). At 15 months of age, prenatal MIA exposure significantly impaired spatial learning function in the LPS mice compared to the CON ones, measured by the distance swam (Figure 3B). The long-term EE treatment improved the spatial learning performance in the LPS-EE group (LPS vs. LPS-EE; Figure 3B).

##### In the memory phase

At 3 months of age, the percentage of distance (Figure 3G) and time (Figure 3H) swam in the LPS group was significantly decreased compared to the CON group, indicating that the spatial memory was declined in the LPS mice. Moreover, short-term EE intervention reversed the memory deficit (LPS vs. LPS-EE; Figure 3G). At 15 months of age, the LPS group showed a decrease in memory consolidation compared to the CON group, as assessed by the percentage of distance and time swam (Figures 3G,H). Meanwhile, the long-term EE exposure restored the memory decline in the LPS-EE group (LPS vs. LPS-EE, Figures 3G,H) and the CON-EE group (CON vs. CON-EE, Figure 3G).

### Serum inflammatory cytokine levels

**Figure 4:**
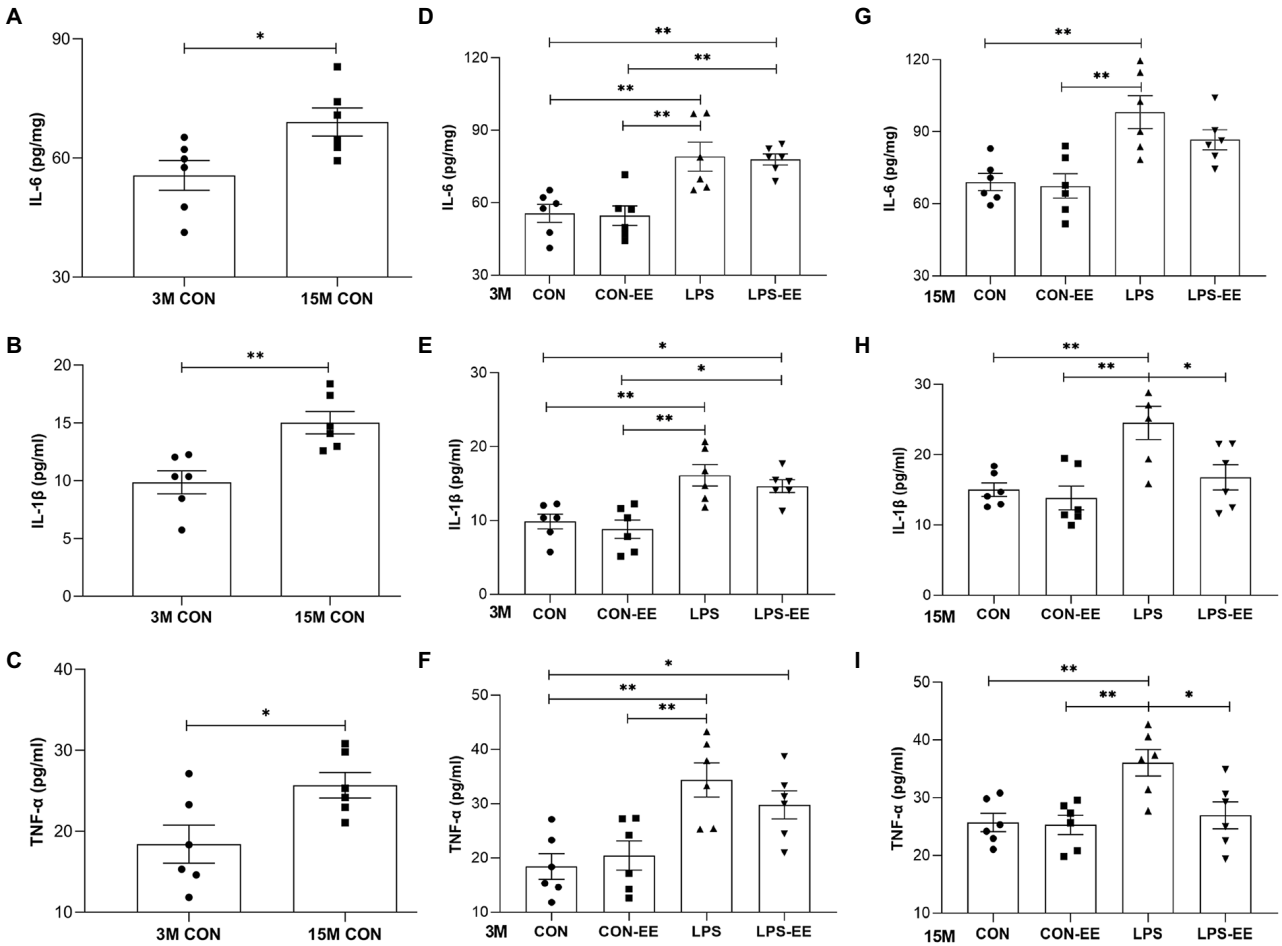
Serum levels of IL-6, IL-1β and TNF-α in mice. Serum levels of IL-6 **(A)**, IL-1β **(B)** and TNF-α **(C)** in the CON group at 3 months (3 M) and 15 months (15 M) of age depicted the effect of age on systematic inflammation. IL-6, IL-1β, and TNF-α levels in the different treatment groups at 3 M **(D-F)** and 15 M **(G-I)**. All data are shown as means ± SEM (*n* = 6 male mice/group). Significance is as follows: *p < 0.05, **p < 0.01. CON, untreated control group; LPS, lipopolysaccharide treatment group; EE, group of mice exposed to environmental enrichment.

Results obtained in the CON mice showed that the serum levels of IL-6, IL-1β, and TNF-α increased with age (Figures 4A–C).

At 3 months of age, embryonic inflammatory exposure activated systemic immune responses accompanied by elevated serum inflammatory cytokine levels (Figures 4D–F). Exposure of mice to EE did not significantly alter serum cytokine concentrations (CON vs. CON-EE; LPS vs. LPS-EE; Figures 4D–F). At 15 months of age, the LPS mice showed a long-term increased inflammatory response compared to the CON ones (Figures 4G–I). Meanwhile, the long-term EE reduced IL-1β and TNF-α levels in the LPS-EE group (LPS vs. LPS-EE; Figures 4H,I).

### Levels of NGPF2 and PSD-95 in the hippocampus

#### NGPF2 mRNA and protein levels

**Figure 5:**
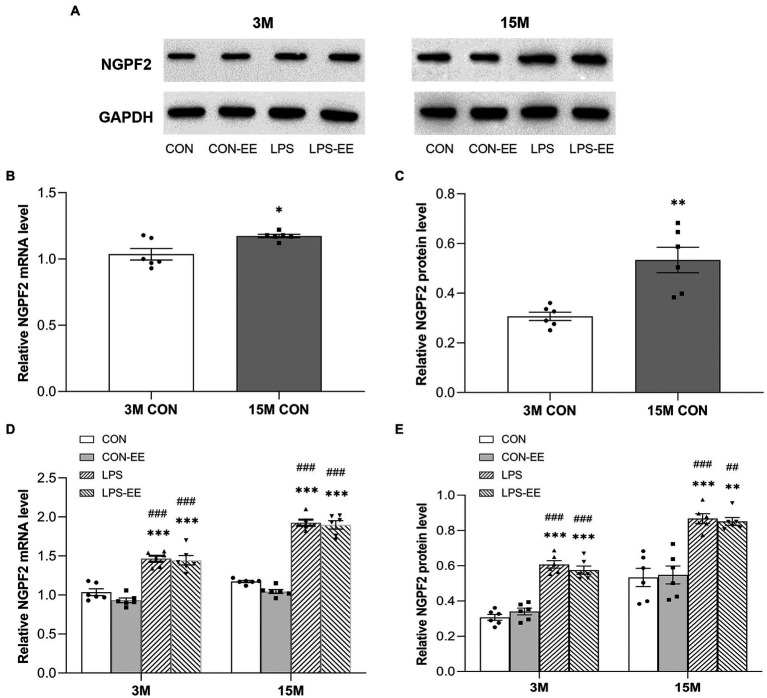
The hippocampal mRNA and protein levels of NGPF2 in CD-1 mice. **(A)** Representative immunoreactive bands for NGPF2 in the different treatment groups at 3 months (3 M) and 15 months (15 M) of age. NGPF2 mRNA and protein levels in the CON group **(B,C)**, and in the CON, CON-EE, LPS and LPS-EE groups **(D,E)** at 3 M and 15 M. All data are provided as mean ± SEM (*n* = 6 male mice/group). ^*^p < 0.05, ^**^p < 0.01, ^***^p < 0.001 compared with the CON group; ^##^*p* < 0.01, ^###^*p* < 0.001 compared with the CON-EE group; CON, untreated control group; LPS, lipopolysaccharide treatment group; EE, group of mice exposed to environmental enrichment.

The levels of NGPF2 mRNA and protein were significantly increased in 15-month-old CON mice compared with 3-month-old mice with the same treatment (Figures 5A–C).

Besides, NGPF2 mRNA and protein levels in the LPS group were significantly higher than those in the CON group, regardless of age (Figures 5D,E). Meanwhile, no significant effects of short or long-term EE intervention were found on NGPF2 mRNA and protein levels (CON vs. CON-EE; LPS vs. LPS-EE).

#### PSD-95 mRNA and protein levels

**Figure 6:**
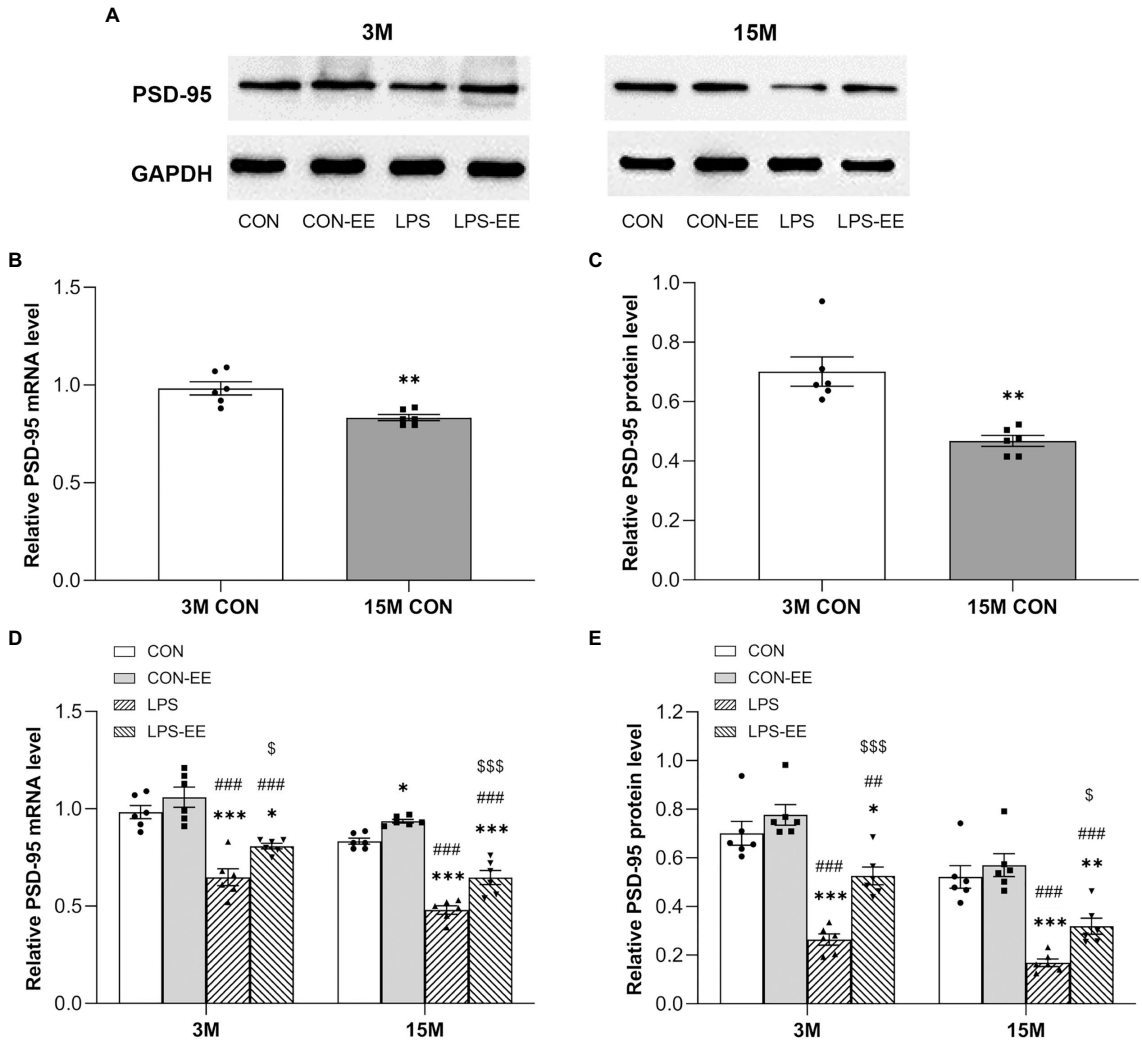
The hippocampal mRNA and protein levels of PSD-95 CD-1 mice. **(A)** Representative immunoblots of PSD-95 in the different groups at 3 months (3 M) and 15 months (15 M) of age. PSD-95 mRNA and protein levels in the CON group **(B,C)**, and in the CON, CON-EE, LPS, and LPS-EE groups **(D,E)** at different ages. All data are shown as mean ± SEM (*n* = 6 male mice/group), ^*^p < 0.05, ^**^p < 0.01, ^***^p < 0.001 compared with the CON group; ^##^*p* < 0.01, ^###^*p* < 0.001 compared with the CON-EE group; ^$^*p* < 0.05, ^$$$^*p* < 0.001 compared with the LPS group; CON, untreated control group; LPS, lipopolysaccharide treatment group; EE, group of mice exposed to environmental enrichment.

In contrast to the mRNA and protein levels of NGPF in the CON group, the mRNA and protein levels of PSD-95 in 15-month-old mice were decreased (Figures 6A–C).

At both 3 and 15 months of age, PSD-95 mRNA and protein levels were downregulated in the LPS group relative to the CON group (Figures 6D,E). Meanwhile, both short- and long-term EE exposure upregulated the levels of PSD-95 mRNA and protein (LPS vs. LPS-EE). In addition, long-term EE alleviated the reduction of *Psd-95* mRNA expression in aged CON mice (CON vs. CON-EE; Figure 6D).

### Correlations between cognitive performance and inflammatory cytokine, NGPF2, and PSD-95

#### Serum inflammatory cytokine

In 3-month-old mice, serum levels of IL-6, IL-1β, and TNF-α were positively correlated with the distance swam during the learning phase in the LPS and LPS-EE groups, and negatively correlated with the percentage of time swam in the target quadrant during the memory phase in the LPS group (Table 2).

**Table 2:** Correlations between performance in MWM trial and serum levels of inflammatory cytokines.

**Cognitive phase**	**Age**	**Group**	**IL-6**	**IL-1β**	**TNF-α**
			***r (p)***	***r (p)***	***r (p)***
Learning distance swam	3 months	CON	0.687 (0.132)	0.544 (0.265)	0.794 (0.060)
		CON-EE	0.566 (0.242)	0.799 (0.056)	0.808 (0.052)
		LPS	0.862 (0.027)*	0.846 (0.034)*	0.818 (0.047)*
		LPS-EE	0.887 (0.019)*	0.849 (0.032)*	0.824 (0.044)*
	15 months	CON	0.784 (0.065)	0.793 (0.060)	0.783 (0.065)
		CON-EE	0.803 (0.054)	0.687 (0.132)	0.789 (0.062)
		LPS	0.876 (0.022)*	0.868 (0.025)*	0.826 (0.043)*
		LPS-EE	0.891 (0.017)*	0.8198 (0.046)*	0.849 (0.032)*
Percentage of distance swam in the target quadrant	3 months	CON	−0.793 (0.060)	−0.508 (0.303)	−0.752 (0.085)
		CON-EE	−0.714 (0.111)	−0.698 (0.123)	−0.752 (0.085)
		LPS	−0.616 (0.193)	−0.752 (0.085)	−0.764 (0.077)
		LPS-EE	−0.775 (0.070)	−0.664 (0.150)	−0.655 (0.158)
	15 months	CON	−0.810 (0.051)	−0.657 (0.156)	−0.775 (0.071)
		CON-EE	−0.694 (0.126)	−0.754 (0.084)	−0.758 (0.081)
		LPS	−0.831 (0.040)*	−0.852 (0.031)*	−0.864 (0.026)*
		LPS-EE	−0.879 (0.021)*	−0.824 (0.044)*	−0.6969 (0.124)
Percentage of time spent swimming in the target quadrant	3 months	CON	−0.750 (0.086)	−0.348 (0.499)	−0.675 (0.141)
		CON-EE	−0.274 (0.600)	−0.629 (0.181)	−0.463 (0.355)
		LPS	−0.832 (0.040)*	−0.821 (0.045)*	−0.860 (0.028)*
		LPS-EE	−0.725 (0.103)	−0.603 (0.205)	−0.645 (0.167)
	15 months	CON	−0.664 (0.151)	−0.254 (0.628)	−0.808 (0.052)
		CON-EE	−0.331 (0.520)	−0.608 (0.200)	−0.472 (0.345)
		LPS	−0.838 (0.037)*	−0.867 (0.026)*	−0.846 (0.034)*
		LPS-EE	−0.810 (0.051)	−0.810 (0.051)	−0.634 (0.176)

*Denotes significant correlation coefficients (*p < 0.05); Significant *p*-values are marked in bold. CON, untreated control group; LPS, lipopolysaccharide treatment group; EE, group of mice exposed to environmental enrichment.

In 15-month-old mice, IL-6, IL-1β, and TNF-α levels positively correlated with the learning distance swam in the LPS and LPS-EE groups and negatively correlated with the memory percentage of distance and time swam in the LPS group (Table 2). Additionally, IL-6 and IL-1β levels showed a negative correction with the memory percentage of distance swam in the LPS-EE group (Table 2).

#### NGPF2

In 3-month-old mice, NGPF2 mRNA and protein levels showed a positive correlation with the learning distance swam in the LPS and LPS-EE groups and a negative correlation with the memory percentage of time swam in the LPS group (Table 3). Furthermore, NGPF2 protein level was negatively correlated with the memory percentage of time swam in the LPS-EE group.

**Table 3:** Correlations between performance in the MWM trial and mRNA/protein levels of NGPF2 and PSD-95.

**Cognitive phase**	**Age**	**Group**	***Ngpf2* mRNA**	**NGPF2 protein**	***Psd-95* mRNA**	**PSD-95 protein**
			***r (p)***	***r (p)***	***r (p)***	***r (p)***
Learning distance swam	3 months	CON	0.268 (0.608)	0.189 (0.720)	−0.736 (0.095)	−0.669 (0.146)
		CON-EE	0.214 (0.683)	0.789 (0.062)	−0.519 (0.291)	−0.611 (0.194)
		LPS	0.874 (0.023)*	0.916 (0.010)*	−0.874 (0.023)*	−0.871 (0.024)*
		LPS-EE	0.841 (0.036)*	0.859 (0.028)*	−0.900 (0.014)*	−0.855 (0.030)*
	15 months	CON	0.101 (0.850)	0.785 (0.064)	−0.738 (0.094)	−0.567 (0.241)
		CON-EE	0.809 (0.051)	0.797 (0.058)	−0.560 (0.247)	−0.450 (0.370)
		LPS	0.860 (0.028)*	0.886 (0.019)*	−0.834 (0.039)*	−0.830 (0.041)*
		LPS-EE	0.867 (0.025)*	0.890 (0.018)*	−0.885 (0.019)*	−0.827 (0.042)*
Percentage of distance swam in the target quadrant	3 months	CON	−0.331 (0.522)	−0.636 (0.175)	0.706 (0.117)	0.441 (0.381)
		CON-EE	−0.772 (0.072)	−0.772 (0.072)	0.664 (0.150)	0.698 (0.123)
		LPS	−0.558 (0.250)	−0.557 (0.250)	0.773 (0.071)	0.236 (0.653)
		LPS-EE	−0.663 (0.152)	−0.396 (0.437)	0.889 (0.018)*	0.543(0.260)
	15 months	CON	−0.734 (0.097)	−0.674 (0.143)	0.300 (0.563)	0.684(0.134)
		CON-EE	−0.617 (0.192)	−0.782 (0.066)	0.79e8 (0.057)	0.497 (0.316)
		LPS	−0.840 (0.036)*	−0.852 (0.031)*	0.878 (0.021)*	0.898 (0.015)*
		LPS-EE	−0.828 (0.042)*	−0.908 (0.012)*	0.877 (0.022)*	0.936 (0.006)**
Percentage of time spent swimming in the target quadrant	3 months	CON	−0.396 (0.437)	−0.273 (0.600)	0.542 (0.266)	0.645 (0.167)
		CON-EE	−0.293 (0.573)	−0.793 (0.060)	0.803(0.055)	0.652 (0.161)
		LPS	−0.835 (0.038)*	−0.885 (0.019)*	0.833 (0.040)*	0.870 (0.024)*
		LPS-EE	−0.702 (0.120)	−0.824 (0.044)*	0.681(0.136)	0.901 (0.014)*
	15 months	CON	−0.564 (0.244)	−0.800 (0.056)	0.761 (0.079)	0.653 (0.160)
		CON-EE	−0.561 (0.247)	−0.742 (0.092)	0.612 (0.197)	0.666 (0.149)
		LPS	−0.747 (0.088)	−0.879 (0.021)*	0.766 (0.076)	0.904 (0.013)*
		LPS-EE	−0.864 (0.027)*	−0.636 (0.174)	0.862 (0.027)*	0.911 (0.012)*

^*^Denotes significant correlation coefficients (*p < 0.05, **p < 0.01); Significant *p*-values are marked in bold. CON, untreated control group; LPS, lipopolysaccharide treatment group; EE, group of mice exposed to environmental enrichment.

In 15-month-old mice, NGPF2 mRNA and protein levels in the LPS and LPS-EE groups positively correlated with learning distance swam and negatively correlated with the memory percentage of distance swam (Table 3). Besides, the memory percentage of time swam was negatively correlated with *Ngpf2* mRNA in the LPS-EE group, as was NGPF2 protein in the LPS group.

#### PSD-95

In 3-month-old mice, PSD-95 mRNA and protein levels negatively correlated with the learning distance swam in the LPS and LPS-EE groups and positively correlated with the memory percentage of swimming time in the LPS group (Table 3). *Psd-95* mRNA level showed a positive correlation with the memory percentage of distance swam in the LPS-EE group, and PSD-95 protein level was positively correlated with the memory percentage of swimming time in the LPS-EE group.

In 15-month-old mice, PSD-95 mRNA and protein levels in the LPS and LPS-EE groups exhibited a negative correlation with the learning distance swam and a positive correlation with the memory percentage of distance swam (Table 3). Furthermore, the memory percentage of time spent swimming positively correlated with *Psd-95* mRNA expression in the LPS-EE group and with PSD-95 protein level in the LPS and LPS-EE groups.

## Discussion

Pregnancy can be complicated by bacterial infections that lead to increases in immune responses in the offspring (Rose et al., 2017; Wang et al., 2020; Su et al., 2022), and bring the neural and behavioral sequelae throughout the lifespan of the offspring (Hambrick et al., 2019; Nelson and Gabard-Durnam, 2020; Van den Bergh et al., 2020). Several studies have shown that there is a gender-dependent effects of learning and memory during aging, while different research demonstrate conflicting results (Wang et al., 2010; Li et al., 2016). Our previous research showed that there were no significant sex differences in spatial learning and memory in normal mice or prenatal MIA-exposed mice at young and old ages (Xiong et al., 2018; Wu et al., 2019; Wang et al., 2020). So were hippocampal levels of inflammation, synaptic proteins, histones, and markers of AD pathology. In this study, we only selected male offspring for research. Here, the results suggested that the LPS mice have elevated levels of systemic inflammation. Meanwhile, prenatal inflammatory exposure accelerated age-associated cognitive dysfunction and changes in *Ngpf2* and *Psd-95* expression in aged mice, whereas EE treatment counteracts some of these influences. Exploration of this potential causal relationship may, to some extent, provide new insights into the mechanisms underlying age-related cognitive impairment.

### Environmental enrichment improved spatial cognitive decline aggravated by embryonic inflammatory exposure

Aging fosters cognitive disabilities (Juan and Adlard, 2019). As described in this study, aging is accompanied by spatial learning and memory decline. Consistent with our results, previous studies had shown age-related decreases in learning and memory starting at 12 months of age in mice (Li et al., 2016; Wang et al., 2020). Moreover, converging lines of evidence indicate that prenatal MIA exposure exacerbates age-related cognitive decline (Wu et al., 2020; Zhang et al., 2020). In the present study, embryonic MIA exposure impaired learning and memory in young mice and accelerated aged-related cognitive deficits in aged mice.

In contrast, multiple studies have demonstrated that EE conveys beneficial effects on cognitive function in mice (Ohline and Abraham, 2019; Crawford et al., 2020; Ismail et al., 2021). Moreover, some detrimental effects of early adverse events can be counteracted by housing mice in cages with more favorable environments (Aghighi Bidgoli et al., 2020; Cordier et al., 2021). Adolescence also represents a critical and sensitive stage in brain development, making it susceptible to environmental stimuli (Wu et al., 2020; Cordier et al., 2021), and therefore, the EE intervention in our study was initiated during adolescence. However, our findings showed that short-term EE (from weaning to 3 months of age) had little effect on cognitive impairment (CON vs. CON-EE; LPS vs. LPS-EE). Notably, long-term EE (from weaning to 15 months of age) exerted a significantly restorative effect on impaired cognition in aged LPS-EE mice (LPS vs. LPS-EE), but not in aged CON-EE ones (CON vs. CON-EE). Other studies have shown that 13 or 14 months of EE exposure improves cognitive deficits in 18-month-old mouse dams exposed to LPS during pregnancy and in their 15-month-old offspring (Wu et al., 2020; Zhuang et al., 2021). These results are similar to ours. In short, our results suggested that long-term EE is necessary to alleviate age-related cognitive decline that is accelerated by embryonic inflammatory exposure.

Additionally, prenatal MIA exposure could also increase the risk of developing psychiatric disorders later in life, such as schizophrenia and autism spectrum disorder (Batinić et al., 2016; Simões et al., 2018). The development of these psychiatric disorders is often accompanied by memory loss during aging. (Roy et al., 2021), which may complicate the results of the measured memory. Our previous work showed that the LPS mice had similar anxieties compared to the same-aged controls at 1, 6, 9, and 14 months of age (Chen et al., 2011; Wang et al., 2020). At 18 months of age, MIA-exposed mice exhibited increased anxieties relative to the same-aged controls (Wang et al., 2020). Overall, these results suggest that prenatal MIA exposure induces an increase in anxiety in an age-dependent manner. In this study, the lack of evaluation of anxiety and depression that may affect memory in mice is a limitation.

### The effects of environmental enrichment on changes in inflammatory cytokine, NGPF2, and PSD-95 accelerated by prenatal inflammatory exposure

Aging is a distinct pro-inflammatory fate (Baker and Petersen, 2018). As shown in this study, the serum inflammatory cytokine levels reflective of systemic inflammatory responses increased with age. In aged mice, the level of glial fibrillary acidic protein, an astrogliosis marker, was also significantly increased in the hippocampus (Rogers et al., 2017).

Several studies suggest that prenatal MIA exposure could alter the immune function in offspring (Hsiao et al., 2012; Rose et al., 2017; Wang et al., 2020). In a MIA model of rhesus monkeys, the offspring were followed until 4 years of age and exhibited increased levels of plasma inflammatory cytokines (Rose et al., 2017). However, little literature has reported whether exposure of embryos to LPS-induced MIA affects systemic immune responses in aged mice. Our data indicated that serum inflammatory cytokine levels were significantly elevated in the LPS group compared to the CON group at both young and old ages. MIA exposure *in utero* also altered neuroimmune modulations in the offspring from embryonic to old age in the mouse hippocampus (Simões et al., 2018; Wang et al., 2020; Guma et al., 2022; Su et al., 2022). For example, previous work from our group has shown that aged LPS mice have increased levels of glial fibrillary acidic protein in the hippocampus relative to age-matched controls (Wang et al., 2020). Briefly, the findings of our group suggested that prenatal MIA exposure enhances age-related increases in inflammatory responses in aged offspring.

NGPF2 has important pathophysiological roles in the CNS (Yoshida et al., 2014). However, there are currently no studies on age-related changes of NGPF2 in the hippocampus. In this study, the results, for the first time, showed an increase in *Ngpf2* expression in the hippocampus of aged mice. Although NGPF2 is at a trivial level in healthy adult mice, it is often upregulated in neuroinflammatory conditions (Kadomatsu et al., 2013; Winkler and Yao, 2014; Ross-Munro et al., 2020). This age-related increase in NGPF2 may somewhat reflect changes in the hippocampal aging process.

The *Ngpf2* gene promoter region contains a binding site for the nuclear factor kappa-B response element, which could be activated by LPS and inflammatory cytokines, such as TNF-α and IL-1β (Cai et al., 2020; Ross-Munro et al., 2020; Domínguez-Rivas et al., 2021). Thus, *Ngpf2* expression is induced after inflammatory injury. For example, NGPF2 protein level was significantly increased after induction of experimental autoimmune encephalitis (Yoshida et al., 2014). Results of our group suggested that prenatal inflammatory exposure increased the inflammation in offspring. As expected, inflammatory exposure *in utero* significantly affected the hippocampal *Ngpf2* expression in this research, with an increase in the LPS mice relative to the CON mice at both young and old ages. Hence, this indicated that prenatal inflammation exposure accelerates age-related upregulation of NGPF2 in aged mice.

Although some studies suggest that EE exposure may improve immune system responses, this possibility has not been widely explored (Keymoradzadeh et al., 2020; Domínguez-Rivas et al., 2021). In this experiment, the effect of EE exposure on serum inflammatory cytokine levels and hippocampal *Ngpf2* expression was minimal (CON vs. CON-EE; LPS vs. LPS-EE).

PSD-95 is a major synaptic protein involved in aging (Savioz et al., 2014; Bustos et al., 2017). In this study, hippocampal *Psd-95* expression decreased with aging, which has been confirmed in other experiments (Rogers et al., 2017; Cały et al., 2021).

Numerous studies have shown that PSD-95 level is reduced in neurodegenerative diseases, like AD (Savioz et al., 2014; Zarate et al., 2021). In young rodents, hippocampal PSD-95 levels have been found to be affected by AD-like pathology or early life stress (Jung and Kim, 2017; Shen et al., 2019; Xiao et al., 2021). Currently, the effects of embryonic inflammatory exposure on hippocampal *Psd-95* expression in aged mice are rarely reported. Here, *Psd-95* expression was significantly decreased in the LPS mice compared to the same-aged controls, especially at old age. In other words, this suggested that prenatal inflammatory exposure enhances age-related downregulation of hippocampal PSD-95. Growing evidence suggests that EE could improve loss of synaptic proteins and impaired synaptic plasticity (Aghighi Bidgoli et al., 2020; Wei et al., 2020; Cordier et al., 2021). PSD-95 is the most abundant scaffolding protein in excitatory postsynaptic density and potently regulates synaptic plasticity (Bustos et al., 2017). Indeed, loss of PSD-95 could cause synaptic dysfunction (Zarate et al., 2021). Our findings showed that both short- and long-term EE treatment significantly restored the decrease in *Psd-95* expression in the mouse hippocampus (LPS vs. LPS-EE). Similarly, EE exposure significantly reversed chronic stress-induced reduction in PSD-95 in the hippocampus of young rats (Shen et al., 2019). Besides, reduced hippocampal PSD-95 was also restored by EE treatment in aged socially isolated mice (Wang et al., 2018). Interestingly, this therapeutic effect was virtually absent in CON-EE mice (CON vs. CON-EE). Thus, our results suggested that EE may serve as a potential intervention to improve impairments in *Psd-95* expression and synaptic function in prenatal MIA-exposed mice.

### Alterations in inflammatory cytokine, NGPF2, and PSD-95 are associated with cognitive dysfunction in aged mice with accelerated aging

Chronic systemic inflammation increases the risk of cognitive impairment (Ashraf-Ganjouei et al., 2020). Specifically, systemic inflammation ultimately activates microglia and astrocytes *via* different pathways, contributing to AD pathology and age-related cognitive decline (Walker et al., 2019). In current study, elevated levels of IL-6, IL-1β, and TNF-α were positively associated with poorer cognition, especially in aged LPS and LPS-EE mice, demonstrating that increased systemic inflammation is closely related to cognitive decline during the accelerated aging. Other studies have shown that patients with AD and mild cognitive deficits tend to have higher levels of peripheral inflammatory cytokines, which are similar to ours.

However, to date, no studies have reported whether *Ngpf2* expression is associated with cognitive impairment in adult mice. Our results showed that cognitive performance was significantly associated with the hippocampal *Ngpf2* expression. Specifically, *Ngpf2* expression was positively correlated with spatial learning and memory impairment in MWM trials, particularly in aged LPS and LPS-EE mice. In traumatic brain injury mice, NGPF2 enhanced neuroinflammation in the brain lesions, leading to worse neurological consequences (Takada et al., 2020). Importantly, NGPF2 could specifically modulate neuroinflammation and influence cognitive changes (Vicente-Rodríguez et al., 2016; Fernández-Calle et al., 2018). In this study, the results demonstrated that an increase in hippocampal NGPF2 appears to be involved in spatial cognition impairment induced by embryonic MIA exposure, especially in aged mice.

As a member of the membrane-associated guanylate kinase family (Savioz et al., 2014), PSD-95 facilitates synaptic maturation by trafficking and anchoring ionotropic glutamate receptors to the postsynaptic membrane (Hambrick et al., 2019). Changes in the level of PSD-95 alter clustering and maintenance of glutamate receptors (Zarate et al., 2021). Thus, PSD-95 plays an essential role in regulating synaptic plasticity, which are fundamental mechanisms that contribute to hippocampal-dependent learning and memory (Savioz et al., 2014; Bustos et al., 2017; Zarate et al., 2021).

However, it is uncertain whether changes in hippocampal *Psd-95* expression are associated with cognitive decline in mice during accelerated aging. As opposed to NGPF2, here, *Psd-95* expression was negatively correlated with spatial learning and memory decline in the aged LPS and LPS-EE mice. Collectively, this suggests that reduced *Psd-95* expression in the hippocampus is associated with cognitive deficits in the mice with accelerated aging. In a young rat model of AD, hippocampal PSD-95 were found to be reduced, with concomitant declines in learning and memory (Xiao et al., 2021). These changes were similar to our 3-month-old LPS mice. A study of natural aging showed that aged mice exhibited reduced hippocampal PSD-95. Interestingly, deficits in long-term potentiation, spatial learning and memory were not observed until 6 months later. This suggested that changes in PSD-95 level do not completely parallel the decline in cognitive and synaptic function during normal aging. In our study, there was also no statistically significant correlation between PSD-95 and cognition in normal aging mice. But when prenatal inflammatory exposure accelerated the decrease in PSD-95, the association was significant, especially in aged mice.

Finally, this study found that long-term EE exposure significantly improved age-related cognitive deficits in the LPS-EE mice. However, the mechanism of this cognitive-improving effect of EE has not yet been elucidated. EE exposure was found to induce an increase in PSD-95 protein level and synaptic plasticity in rodent’s hippocampus, with concomitant improvement in cognitive deficits (Jung and Kim, 2017; Shen et al., 2019). Moreover, *Psd-95* expression is downregulated in hippocampus with aging, AD, and other pathologies (Savioz et al., 2014; Rogers et al., 2017; Xiao et al., 2021). Thus, *Psd-95* expression may counteract aging or pathological processes (Savioz et al., 2014). Because the effects of EE intervention on *Ngpf2* expression and serum inflammatory cytokine levels were minimal, we speculate that EE exposure repaired cognitive impairment by increasing *Psd*-95 expression and thereby improving synaptic plasticity. Indeed, our results confirmed that EE exposure significantly restored the decrease in hippocampal *Psd-95* expression in the LPS-EE mice. Collectively, changes in hippocampal *Ngpf2* and *Psd-95* expression were associated with age-dependent cognitive decline in mice with accelerated aging, and long-term EE may ameliorate this cognitive decline by improving *Psd-95* expression.

In summary, prenatal MIA exposure accelerates age-related declines in spatial learning and memory and exacerbates age-related changes in hippocampal *Ngpf2* and *Psd-95* expression and peripheral inflammatory cytokine levels in offspring mice. These biochemical changes were closely correlated with impaired spatial cognition, particularly during “pathological” aging. Furthermore, long-term EE exposure attenuated these cognitive declines, possibly by enhancing *Psd-95* expression. Therefore, EE exposure and targeted modulation of neuroinflammation may represent a potential therapeutic strategy for age-related cognitive decline. Limitations in the present study include not investigating the level of hippocampal inflammation, treatment effects on *Ngpf2* and *Psd-95* expression in different subregions of the hippocampus, and sex differences. In the future, we will explore the effects of embryonic exposure to maternal inflammation on NGPF2-related signaling pathways, neuroinflammatory signaling pathways, and synaptic plasticity in mice.

## Data availability statement

The raw data supporting the conclusions of this article will be made available by the authors, without undue reservation.

## Ethics statement

The animal study was reviewed and approved by the Experimental Animal Ethics Committee of Anhui Medical University.

## Author contributions

M-ZN and Y-MZ conceived and designed the study, performed the experiments, and drafted the manuscript. Q-TW and YL conducted the experiments. JC and B-LL conducted the behavioral tests and collected the data. Z-ZZ performed the statistical analyses. G-HC and X-WL revised the manuscript. All authors contributed to the article and approved the submitted version.

## Funding

This work was financially supported by the National Natural Science Foundation of China (81370444 and 81671316), Scientific Research Fund Project of Hunan Provincial Health Commission (20200497), and Natural Science Foundation of Hunan Province of China (2021JJ70040).

## Conflict of interest

The authors declare that the research was conducted in the absence of any commercial or financial relationships that could be construed as a potential conflict of interest.

## Publisher’s note

All claims expressed in this article are solely those of the authors and do not necessarily represent those of their affiliated organizations, or those of the publisher, the editors and the reviewers. Any product that may be evaluated in this article, or claim that may be made by its manufacturer, is not guaranteed or endorsed by the publisher.
