# Supplementary material for: Environmental enrichment improves declined cognition induced by prenatal inflammatory exposure in aged CD-1 mice: Role of NGPF2 and PSD-95
Source: Front Aging Neurosci. 2022 Nov 21;14:1021237. doi: 10.3389/fnagi.2022.1021237 (PMC9720164; doi:10.3389/fnagi.2022.1021237)
Supplement: Supplementary file 1 [file Table_1.DOCX]

| **Supplementary Table 1\| Results of different treatment groups of mice at 3 months of age and 15 months of age in the MWM test** | | | | | | | | | |
| --- | --- | --- | --- | --- | --- | --- | --- | --- | --- |
| **Cognitive parameters** | **3 months of age** | | | |  | **15 months of age** | | | |
|  | **CON** | **CON-EE** | **LPS** | **LPS-EE** |  | **CON** | **CON-EE** | **LPS** | **LPS-EE** |
| Swimming velocity (means; cm/s) | 27.45 | 29.98 | 36.32 | 34.46 |  | 26.70 | 25.44 | 34.45 | 26.59 |
| Distance swam (means; m) | 4.573 | 5.215 | 8.356**^***^** ^##^ | 7.464**^**^**^#^ |  | 7.782 | 6.880 | 10.91 **^**^** ^###^ | 7.408^$$$^ |
| Escape latency (s) | 17.89 | 17.92 | 22.45 | 22.43 |  | 31.97 | 28.99 | 32.88 | 30.60 |
| Distance percentage (means ± SEM; %) | 52.93±1.35 | 56.20±2.12 | 41.08±0.91**^***^**^###^ | 46.70±0.92**^*^** ^## $^ |  | 39.15±1.20 | 46.06±1.28 **^*^** | 29.26±1.90**^**^**^###^ | 36.74±2.21^## $^ |
| Time percentage (means ± SEM; %) | 40.80±1.95 | 42.17±1.675 | 28.06±1.86**^***^**^###^ | 33.14±1.82**^*^** |  | 34.42±1.40   \|  \| 2.423 \| 1.455 \| 1.279 \| \| --- \| --- \| --- \| --- \| | 38.14±2.42 | 18.64±1.46**^***^**^###^ | 27.22±1.28**^*^** ^## $$^ |
| ^*^*P* < 0.05, ^**^*P* < 0.01, ^***^*P* < 0.001 compared with the CON group; ^#^*P* < 0.05, ^##^*P* < 0.01, ^###^*P* < 0.001 compared with the CON-EE group; ^$^*P* < 0.05, ^$$^*P* < 0.01, ^$$$^*P* < 0.001 compared with the LPS group; | | | | | | | | | |
